# Management of septic shock: a protocol-less approach

**DOI:** 10.1186/s13054-015-0968-8

**Published:** 2015-06-19

**Authors:** Jorge L. Cabrera, Michael R. Pinsky

**Affiliations:** Department of Critical Care Medicine, University of Pittsburgh, 642A Scaife Hall, 3550 Terrace Street, Pittsburgh, PA 15261 USA; Department of Critical Care Medicine, University of Pittsburgh, 606 Scaife Hall, 3550 Terrace Street, Pittsburgh, PA 15261 USA

## Abstract

**Citation:**

ProCESS Investigators, Yealy DM, Kellum JA, Huang DT, Barnato AE, Weissfeld LA, Pike F, Terndrup T, Wang HE, Hou PC, LoVecchio F, Filbin MR, Shapiro NI, Angus DC. A randomized trial of protocol-based care for early septic shock. N Engl J Med. 2014; 370:1683–93.

**Background:**

In a single-center study published more than a decade ago involving patients presenting to the emergency department with severe sepsis and septic shock, mortality was markedly lower among those who were treated according to a 6-h protocol of early goal-directed therapy (EGDT), in which intravenous fluids, vasopressors, inotropes, and blood transfusions were adjusted to reach central hemodynamic targets including central venous pressure, central venous oxygen saturation, and indirect estimates of cardiac output, than among those receiving usual care.

**Methods:**

*Objective*: The objective was to determine whether these EGDT findings were generalizable and whether all aspects of the EGDT protocol were necessary to achieve those outcomes.

*Design*: A multicenter randomized three-arm controlled trial.

*Setting*: Thirty-one academic emergency departments in the United States.

*Subjects*: Patients older than 18 years of age presenting to the emergency department with septic shock.

*Intervention*: Patients were assigned to one of three groups for 6 h of resuscitation: protocol-based EGDT as defined by River and colleagues; protocol-based standard therapy that did not require the placement of a central venous catheter, administration of inotropes, or blood transfusions; and usual care which mandated no specific monitoring or management approaches.

*Outcomes*: The primary end point was 60-day in-hospital mortality. Also tested sequentially was whether protocol-based care (EGDT and standard therapy groups combined) was superior to usual care and whether protocol-based EGDT was superior to protocol-based standard therapy. Secondary outcomes included longer-term mortality and the need for organ support.

**Results:**

A total of 1,351 patients were enrolled, of whom 1,341 were evaluable due to patient/family request: 439 were randomly assigned to protocol-based EGDT, 446 to protocol-based standard therapy, and 456 to usual care. Resuscitation strategies differed significantly with respect to the monitoring of central venous pressure and central venous oxygen and the use of intravenous fluids, vasopressors, inotropes, and blood transfusions. By 60 days, there were 92 deaths in the protocol-based EGDT group (21.0 %), 81 in the protocol-based standard therapy group (18.2 %), and 86 in the usual care group (18.9 %) (relative risk with protocol-based therapy versus usual care, 1.04; 95 % confidence interval, 0.82 to 1.31; *P* = 0.83; relative risk with protocol-based EGDT versus protocol-based standard therapy, 1.15; 95 % CI, 0.88 to 1.51; *P* = 0.31). There were no significant differences in 90-day mortality, 1-year mortality, or the need for organ support.

**Conclusions:**

In a multicenter trial conducted in the tertiary care setting, protocol-based resuscitation of patients in whom septic shock was diagnosed in the emergency department did not improve outcomes.

## Commentary

Septic shock is the leading cause of mortality in patients admitted to the ICU [1, 2]. In the United States alone there are over 750,000 cases of severe sepsis and septic shock each year [3]. Although the mortality associated with severe sepsis and septic shock 12 years ago was ~30 %, epidemiological studies show that sepsis short-term mortality has steadily decreased to just over 18 % today [4–6]. In a 2001 single-center randomized controlled trial published by Rivers and colleagues, however, early goal-directed therapy (EGDT) versus standard therapy significantly lowered mortality (30.5 % versus 46.5 %) [7]. Primarily based on this single-center trial, both the Society of Critical Care Medicine and the European Society of Intensive Care Medicine published guidelines in 2004, later updating them in 2008 and 2012, all recommending protocol-based care for severe sepsis, including EGDT. Importantly, a fundamental component of the EGDT guidelines is the insertion of a central venous catheter (CVC) to monitor central venous pressure and central venous oxygen saturation (ScvO_2_) to guide the use of intravenous fluids, as well as the use of vasopressors, packed red blood cell transfusions, and dobutamine to achieve prespecified mean arterial pressure, central venous pressure, and ScvO_2_ goals. However, questions remained as to which elements of EGDT accounted for this reduction in mortality and whether or not a simpler more pragmatic approach could yield similar outcome benefits.

In the ProCESS trial, investigators at 31 US academic emergency departments randomized 1,351 patients (1,341 enrolled), age ≥18 years, presenting with septic shock to one of three arms for 6 h of resuscitation: protocol-based EGDT; protocol-based standard therapy not requiring the insertion of a CVC; and usual care (wild-type). In the protocol-based EGDT group, the resuscitation team followed a protocol which mimicked that used by Rivers and colleagues (CVC insertion, monitoring of central venous pressure and ScvO_2_, intravenous fluids, pressors, inotropes, and blood transfusions) [7]. The protocol-based standard therapy called for the administration of intravenous fluids to be titrated to achieve systolic blood pressure and Shock Index (defined as the ratio of heart rate to systolic blood pressure) parameters while monitoring for clinical symptoms of fluid overload. Importantly, the use of a CVC was not required unless needed for venous access and patients received packed red blood cell transfusions only if the hemoglobin level was <7.5 g/dl. In the usual care group, bedside providers directed all care; and since there was no protocol to follow, all decisions were left to the treating physician.

The primary outcome of the study was all-cause in-hospital mortality at 60 days. The 60-day in-hospital mortality for the combined protocol-based groups (19.5 %) did not differ significantly from that in the usual care group (18.2 %) (relative risk, 1.04; 95 % confidence interval, 0.82 to 1.31; *P* = 0.83), nor did mortality differ significantly when the groups were compared separately. Likewise, there were no significant differences in 90-day mortality or in the time to death up to 90 days and 1 year amongst the three groups. Importantly, both the protocol-based standard care and the usual care groups received fewer CVCs than the protocol-based EGDT group (56.5 %, 57.9 %, and 93.2 % respectively). These data suggest that in the setting of a well-staffed tertiary care emergency department that closely assesses the clinical status of patients in septic shock, using a defined protocolized approach did not alter outcome.

The obvious strengths of this study are that it was a multicenter randomized controlled trial not a single-center trial, and that with 1,341 enrollees this study was five times the size of Rivers and colleagues’ trial. In addition, the investigators in the ProCESS trial thoroughly monitored protocol adherence.

As with all clinical trials, there were some specific limitations. The investigators excluded pregnant patients and those with advanced AIDS from the study, two groups of patients that often present with severe infections. The results therefore do not aid the emergency medicine physician or intensivist when managing these two important subgroups. Similarly, because the study was carried out in tertiary academic emergency departments, concerns remain as to its applicability in community emergency departments, urgent care centers, and on general medical wards. This concern is highlighted by the fact that, even in the usual care group, patients received on average 2.1 l intravenous fluids prior to randomization and another 2.3 l from randomization to the 6-h mark. Are most community emergency departments in the United States providing 4.4 l intravenous fluids in the first 6 to 8 h after the detection of septic shock to their patients? The answer to this question remains unknown.

It is worth mentioning that this study does not undermine the one published by Rivers and colleagues, which brought a consciousness of early recognition and aggressive management to patients with septic shock. Indeed, a recent retrospective analysis of severe sepsis mortality underscores the progressive reduction in sepsis mortality over the past 12 years without the introduction of a single new treatment other than protocolized care centered around early recognition and management of severe sepsis [6]. Those principles are still valid today. This is also apparent in the ProCESS trial, where the mortality in all groups was much lower than that seen in Rivers and colleagues’ treatment or control arms. Importantly, patients in the ProCESS trial were rapidly identified as having severe sepsis/septic shock and treated with a bolus of intravenous fluids and intravenous antibiotics prior to enrollment in the resuscitation arms. Thus, while this trial suggests some components of the EGDT algorithm are unnecessary (CVC insertion, continuous ScvO_2_ monitoring, dobutamine, and transfusion triggers for hematocrit <30 %), it does substantiate that prompt recognition coupled with resuscitation, administration of antibiotics, and source control are the cornerstones for the treatment of septic shock.

So are we now ready to adapt a protocol-less approach to the management of patients with septic shock? Some specific conclusions can be drawn from the ProCESS trial that are further substantiated by the recent publications of two other sepsis trials – the Australasian Resuscitation in Sepsis Evaluation (ARISE) trial [8] and the Protocolised Management in Sepsis (ProMISE) trial [9] – and in general confirm the finding of no difference in patient-centered outcomes between EGDT and usual care. First, continuous ScvO_2_ monitoring is unwarranted as a universal requirement [10]; this is supported by the fact that in the ProCESS trial the ICU admission rate was approximately 5 % less for the two non-EGDT groups without this monitoring technique than for the protocol-based EGDT group. Similarly, CVC insertion should be individualized, as should decisions about the transfusion threshold. It remains to be seen what the cost-saving implications will be for the healthcare system moving forward.

## Recommendation

Based on the results of this large multicenter randomized controlled trial, patients presenting with septic shock at academic emergency departments in the USA can be safely managed with a protocol-less approach that focuses on patient response to routine resuscitation management, early antibiotic use, and close observation.

## Abbreviations

CVC: Central venous catheter
EGDT: Early goal-directed therapy
ScvO_2_: Central venous oxygen saturation
